# Restricted Spatial Windows of Visibility in Myalgic Encephalomyelitis (ME)

**DOI:** 10.3390/vision2010002

**Published:** 2018-01-17

**Authors:** Nadia S. Ahmed, Irene Gottlob, Frank A. Proudlock, Claire V. Hutchinson

**Affiliations:** Department of Neuroscience, Psychology and Behaviour, College of Life Sciences, University of Leicester, Leicester LE1 7RH, UK

**Keywords:** contrast sensitivity, myalgic encephalomyelitis, chronic fatigue syndrome, spatial vision, visual psychophysics, windows of visibility

## Abstract

Myalgic encephalomyelitis (ME) is a devastating disorder marked by debilitating fatigue. It not well understood and its diagnosis is controversial. It is very important therefore that significant clinical features are investigated. Visual symptoms in ME represent a group of distinct, quantifiable, clinical features that could significantly improve diagnosis and provide insights into underlying pathology. The purpose of the present study was therefore to explore the effect of ME on spatial windows of visibility using the spatial contrast sensitivity function. Contrast sensitivity was determined for stationary luminance-defined sinusoidal gratings spanning a five-octave range of spatial frequencies (0.5 to 16 c/deg) in a group of 19 individuals with ME and a group of 19 matched (age, gender) controls. Compared to controls, the ME group exhibited a restricted spatial window of visibility for encoding stimulus contrast. This was characterised principally by a contrast sensitivity deficit at lower spatial frequencies and a narrower bandwidth. Our findings suggest that contrast sensitivity deficits may represent a visual marker of ME, and be indicative of abnormal visual processing at the level of the retina and in the cortical and subcortical visual pathways.

## 1. Introduction

Myalgic encephalomyelitis (ME) is a debilitating disorder, affecting over 250,000 people in the UK [1]. It represents a substantial disease burden on sufferers, their families, the health service and economy. Marked by debilitating fatigue, it is not well understood and its diagnosis is controversial. There are a variety of case definitions for ME [2,3,4,5], though to date none have been formally operationalised. Diagnosis is based on categorising the symptoms reported by patients and differentiating them from symptoms of other fatigue-related illnesses. It is a diagnosis of exclusion, because there is currently no specific diagnostic test for ME. This is problematic due to the number of symptoms overlapping with other illnesses, and the reliance on patients to report their symptoms accurately. The result is that ME is commonly misdiagnosed, often as depression [6]. It is very important therefore that significant clinical features are investigated. 

Visual symptoms in ME represent a group of distinct, quantifiable, clinical features that could significantly improve diagnosis, provide insights into underlying pathology and represent a candidate for treatment, thereby improving the everyday lives of patients. Commonly-reported visual symptoms include photosensitivity, difficulty focussing on images, blurring of images, halos around images, poor depth perception, pain in the eyes, impaired visual attention, increased susceptibility to pattern glare, and vision-related headaches [7,8,9,10,11]. A number of visual problems have also been identified objectively using experimental measures and include, reduced visual accommodation and poor binocular vision [12,13], increased susceptibility to pattern-glare [14], inaccurate eye movements [15], and impaired visual attention, particularly on tasks on which performance relies upon the ability to detect and/or identify an object whilst ignoring irrelevant visual distractors (selective attention) [16]. Visual symptoms are a pervasive part of the condition, exacerbate other symptoms, and affect the ability to carry out every day tasks [7,8,9]. Indeed, some studies report that up to 25% of those suffering from ME reduce the frequency of driving or stop driving completely due to the vision-related problems they experience [7].

In spite of reported problems related to the perception of even elementary spatial information, the effects of ME on spatial vision remain relatively unexplored and unquantified. The purpose of the present study was therefore to explore how ME affects spatial windows of visibility using the spatial contrast sensitivity function. The human contrast sensitivity function provides a measure of the range of spatial detail that is visible (resolvable) to the visual system and the relative sensitivity to stimulus contrast within this range. Changes in contrast sensitivity are well documented in ageing [17] and are evident in a range of retinal diseases [18]. They are also present in neurodegenerative diseases such as Parkinson’s disease [19] and in inflammatory autoimmune diseases such as multiple sclerosis [20,21,22]. Contrast sensitivity deficits can be present even when there is no detectable impairment in visual acuity. They provide a sensitive clinical measure of visual function and can indicate abnormal visual processing at the level of the retina and in the cortical and subcortical visual pathways. 

In the present study, contrast sensitivity was determined for stationary luminance-defined sinusoidal gratings spanning a five-octave range of spatial frequencies (0.5 to 16 c/deg) in a group of ME patients and controls.

## 2. Materials and Methods

### 2.1. Participants

20 ME patients and 20 matched (age, gender, education) controls were originally recruited to the study. One patient only completed two of the six spatial frequencies in the contrast sensitivity experiment so both the patient and his matched control were removed from the contrast sensitivity analysis. All patients had a medical diagnosis of ME, confirmed with the DePaul Symptom Questionnaire [23]. Only participants who fulfilled these criteria were included. Participants had no history of ocular disease. Monocular visual acuity at near and distance was in the normal range for all participants included in the study. A two (group: patients, controls) by two (eye: left, right) mixed, repeated measures ANOVA confirmed that there were no significant differences in corrected visual acuity at near between patients and controls (F_1,38_ = 1.113; *p* = 0.298) or between the two eyes (F_1,38_ = 0.538; *p* = 0.468). A 2 (group: patients, controls) by 2 (eye: left, right) ANOVA on the distance acuity data also confirmed that there were no significant differences in corrected visual acuity at distance between patients and controls (F_1,38_ = 0.2808; *p* = 0.102) or between the two eyes (F_1,38_ = 0.229; *p* = 0.635). Ethical approval was granted by the University of Leicester. All experimental methods adhered to the tenets of the Declaration of Helsinki. Informed consent was obtained before the study commenced.

### 2.2. Apparatus and Stimuli

Gratings subtended six deg (horizontally & vertically) at a viewing distance of 139 cm and were generated using a Macintosh G4 and presented on a Sony Trinitron CRT monitor with an update rate of 75 Hz using the C programming language. The monitor was gamma-corrected using a spot photometer (LS-100, Konica Minolta, Tokyo, Japan) and look-up-tables (LUT). For precise control of luminance contrast the number of intensity levels available was increased from 8 to 14 bits using a Bits++ attenuator (Cambridge Research Systems, Cambridge, UK). The mean luminance of the display was ~44 cd/m^2^ and the monitor was the only light source. Total stimulus presentation duration was 853 ms and the luminance contrast of the sinusoidal waveform was smoothed on and off by half a cycle of a raised cosine lasting 170 ms. In a similar manner the sinusoidal waveform was spatially windowed in the horizontal dimension according to a half cycle of a raised cosine function with a half-period of 1.2 deg. This was done to minimise the presence of spatial and temporal transients. 

The luminance contrast of the pattern could be varied according to the following equation:

Luminance contrast = (L_max_ − L_min_)/(L_max_ + L_min_), (1)

where L_max_ and L_min_ are the maximum and the minimum luminances of the grating, in the range 0–1. 

### 2.3. Procedure

Contrast threshold measurements were taken across a five-octave range of spatial frequencies (0.5 to 16 c/deg) using a single-interval, forced-choice procedure. On each trial, participants were presented with a fixation cross, followed by the presentation of the grating, upon which they were required to judge its orientation (vertical or horizontal). Participants were allowed a short practice run and the testing was performed in the dark. The luminance contrast of the test stimulus was varied from trial to trial according to a modified three-down, one-up staircase designed to converge on the contrast corresponding to 79.4% correct [24,25]. At the beginning of each run of trials the contrast of the test pattern was initially set to a suprathreshold level (typically ~6 dB above threshold) and the initial staircase step size was chosen to be half this value. On subsequent reversals the step size was halved and testing was terminated after a total of 16 reversals. Threshold estimates were taken as the mean of the last 4 reversals in each staircase. Each observer completed two staircases per condition and a mean was taken. The order of testing was randomised. Contrast thresholds were converted to contrast sensitivity (1/contrast threshold). 

## 3. Results

**Figure 1:**
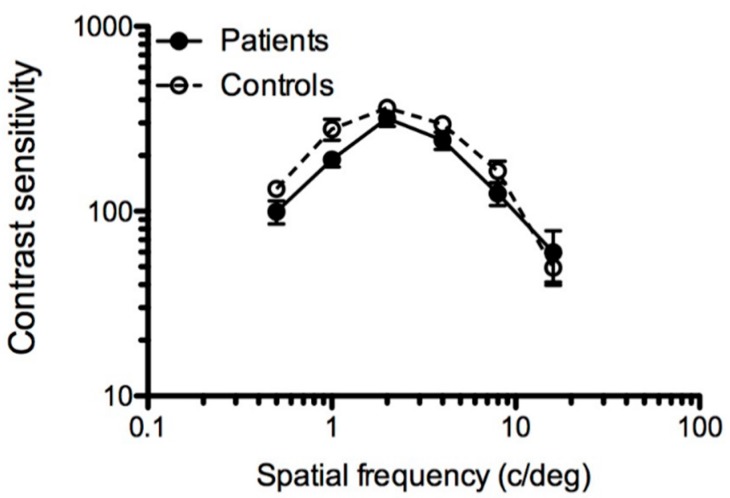
Spatial contrast sensitivity functions (0.5 to 16 c/deg) for the myalgic encephalomyelitis (ME) group (closed symbols) and controls (open symbols). Error bars represent ±1 S.E.M.

Mean contrast sensitivity functions for each group are shown in Figure 1 and exhibited the expected bandpass character. Overall, the contrast sensitivity function was somewhat depressed and appeared more narrowband for the ME group, suggesting a restricted window of visibility in ME, compared to controls. A six (spatial frequency: 0.5, 1, 2, 4, 8, 16 c/deg) by two (group: patients, controls) mixed, repeated measures ANOVA yielded the following statistically significant differences: an effect of spatial frequency (F_5,180_ = 31.650; *p* < 0.001) and group (F_1,36_ = 7.081; *p* < 0.05) [note that three of the expected 228 values (6 spatial frequencies × 19 participants × 2 groups) were missing: two for controls, and one for a patient. For the repeated measures ANOVA, the three missing data points were replaced by the mean for that group and condition (replacement by mean)]. *t*-Tests showed that contrast sensitivity at the lowest spatial frequencies (0.5 and 1 c/deg) was significantly worse in patients compared to controls (0.5 c/deg: t_36_ = 0.2.076; *p* < 0.05; 1 c/deg: t_36_ = 2.214, *p* < 0.05).

To quantify more precisely the spatial window of visibility for each participant group, contrast sensitivity at each spatial frequency was converted to relative sensitivity (dB) using the following equation:

(2) $$Relative\;sensitivity\;\left(dB\right)=\;20\times \log_{10}\left(\frac{Si}{Smax}\right)$$

where *Smax* corresponds to the maximum contrast sensitivity and *Si* is the contrast sensitivity at each spatial frequency. 

**Figure 2:**
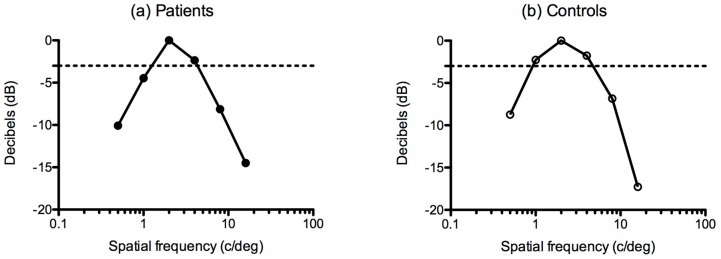
Relative sensitivity (dB) for (**a**) the myalgic encephalomyelitis (ME) group and (**b**) controls derived from Equation (1). Analysis was performed separately on the mean contrast sensitivity data for each group. Dashed lines represent—3 decibels from peak sensitivity (at which sensitivity drops to half its maximal power).

This conversion allowed the determination of lower and upper corner frequencies for each group (Figure 2). These values correspond to the spatial frequencies at which sensitivity dropped to half its maximal power, taken at 3 decibels below peak sensitivity. Corner frequencies were used to calculate the bandwidth (half width, half height) of the spatial window of visibility for the ME group and controls, as follows:

(3) $$Bandwidth\;\left(octaves\right)=\log_{2}\left(\frac{f_{2}}{f_{1}}\right)\;$$

where $f_{1}$ is the lower corner frequency and $f_{2}$ is the upper corner frequency.

Figure 2 shows relative sensitivity, calculated using the group data presented in Figure 1. Additionally, relative sensitivity was calculated for each individual participant, allowing the calculation of lower and upper corner frequencies, and corresponding bandwidths, from which mean values, with corresponding standard errors, for the ME group and controls are given in Table 1. *t*-Tests showed that lower corner frequencies were significantly higher in the ME group compared to controls (t_36_ = 2.556; *p* < 0.05). There were no differences between upper corner frequencies between groups (t_36_ = 0.822; *p* = 0.416). Lower corner frequencies in the ME group contributed to narrower overall bandwidths (t_36_ = 2.82; *p* < 0.05). 

## 4. Discussion

Here, we have shown that a group of ME patients exhibited a restricted spatial window of visibility for stimulus contrast compared to a control group. This was characterised principally by a contrast sensitivity deficit at lower spatial frequencies. These findings suggest that contrast sensitivity deficits may represent a visual marker of ME, and possibly be indicative of abnormal visual processing at the level of the retina and in the cortical and subcortical visual pathways. 

The findings presented here add to a growing literature demonstrating that vision-related problems represent a measurable class of symptoms that are commonly reported by patients with ME. Self-report studies have highlighted the existence of ME-related visual problems which include blurred vision, diplopia, floaters, photophobia, dry, gritty and tired eyes, ocular burning and non-specific eye pain, poor oculomotor control, poor depth perception, spots, lights and halos in the visual field, and vision-related headaches and migraines [7,8,9,10]. Other studies have revealed abnormalities of the pre-ocular surface [12] and vascular pathology in the eye [26]. There is also evidence for a significantly higher distribution of exophoria, lower functional vergence (near and far), a further point of convergence, and lower tear secretion and break up time in ME patients, compared to healthy controls [27], reduced accommodation [12,13], impaired anti-saccadic and smooth pursuit eye movements [15] deficits in visual attention (determined using visual cueing, visual search & selective visual attention tasks) [16] and increased susceptibility to pattern-related visual stress [11,14].

Although we did not directly address the relationship between susceptibility to migraine and contrast sensitivity in the present study, it is of note that some other studies have found a relationship between migraine and contrast sensitivity deficits (see O’Hare and Hibbard [28] for a recent review). Of particular relevance to the contrast sensitivity deficits reported here is a study by Benedek et al. [29], who found a contrast sensitivity deficit at low spatial frequencies in a group of migraine sufferers compared to controls. It is well-documented that people with ME find some visual patterns are uncomfortable [11,14] and report an increased susceptibility to photosensitivity and migraine [7,8,9,10]. As such, future studies examining the relationship between vision-related headaches and migraines in ME and contrast sensitivity deficits is of potential importance and may provide some insight in to the effects of ME on the neurophysiology of the visual system.

Our findings bear similarity to contrast sensitivity deficits recently reported for multiple sclerosis (MS) [22]. MS is an inflammatory, neurodegenerative autoimmune disease [30]. Like people with ME, MS patients exhibit a wide range of visual symptoms. Optic neuritis is common [31] along with reports of loss of vision, colour vision disturbances, pain in the eyes, blurred vision, and visual fatigue [30,31]. A number of studies have examined contrast sensitivity in MS, and although the spatial frequency specificity varies between studies, there is agreement that poor contrast sensitivity is associated with the condition [20,21,22]. MS and ME share phenomenology and neuroimmune characteristics [32]. The onset of ME has been associated with infections and autoimmune disorders and, like MS patients, people with ME exhibit a range of immune abnormalities indicating dysregulation of the immune system [33]. As such, contrast sensitivity deficits, as well as representing a possible clinical marker of ME, may also lend weight to the notion that ME, like MS, is a disorder of the autoimmune system.

There are some caveats concerning the clinical utility of the type of contrast sensitivity testing used in the present study. Measuring the full spatial frequency dependent contrast sensitivity function using traditional psychophysical techniques, as we have done in the present study, is typically very time-consuming, with the result that the clinical utility of this type of testing method is limited. Contrast sensitivity can also be determined using letter charts in which the contrast of large letters decreases on each horizontal line. The most commonly used contrast letter chart is the Pelli-Robson Chart [34]. Pelli-Robson Charts do not provide as sensitive a measure of contrast sensitivity as traditional psychophysical contrast thresholding procedures, but they have the advantage of being much faster and easier to administer in a clinical setting. Furthermore, it is difficult to quantify the spatial frequency content of contrast letter charts because letter identification depends on the component spatial frequencies reaching threshold [35]. The question remains therefore whether they provide a sufficiently sensitive measure of contrast sensitivity to identify deficits in the early stages of disease and/or whether they can detect small changes in contrast sensitivity over time (i.e., before and after treatment). We followed up with the Pelli–Robson Chart, the spatial-frequency specific contrast sensitivity deficits reported here, and found that our ME group performed well and at equivalent levels to controls.

In terms of the clinical value of determining contrast sensitivity in ME, the psychophysical methods we have used in the present study are likely to be too time-consuming and the Pelli–Robson Test is not sufficiently sensitive. Recent developments in Bayesian adaptive procedures which provide quick measurement of the contrast sensitivity function [36,37] are promising in this context. These procedures have been shown to achieve good agreement with contrast sensitivity functions determined using conventional psychophysical methods (such as those employed in the present study) in normal and clinical vision [38]. Such procedures have been successfully implemented for measuring of contrast sensitivity in amblyopia [39]. They have also been suggested as a means of measuring the contrast sensitivity functions of low vision patients [40].

One notable limitation of our study concerns our ME sample. Our visual testing protocols required carefully calibrated equipment. As such, we were only able to test ME patients who were able to travel to the university to take part in the study. Whilst the participants in our study were significantly affected by ME, we were unable to measure contrast sensitivity in those most severely affected, e.g., those who are bedbound by the condition. As a result, our findings are likely to underrepresent deficits in this group. 

In summary, because there is no established cause of ME, no conclusive tests to determine its presence and no definitive outward signs that set it apart from other disorders, clinicians must rely on patients’ self-perceptions and reports. As such, identifying distinct clinical features of ME is an important issue. Here, we have reported contrast sensitivity deficits in a group of ME patients. We propose that these findings, along with other visual problems experienced by people with ME, may have implications for diagnosis and may provide some insights into its aetiology. They also raise a number of important questions that warrant further study. 

## Figures and Tables

**Table 1:** Mean lower and upper corner frequencies, and bandwidths (±1 SE) of the contrast sensitivity functions for the ME group and controls.

	Lower Corner Frequency (c/deg)	Upper Corner Frequency (c/deg)	3 dB Bandwidth (octaves)
ME Group	1.44 (0.173)	4.53 (0.465)	1.57 (0.164)
Controls	0.99 (0.041)	5.09 (0.502)	2.23 (0.165)
